# Sex Influence on Heart Failure Prognosis

**DOI:** 10.3389/fcvm.2020.616273

**Published:** 2020-12-21

**Authors:** Andrea Postigo, Manuel Martínez-Sellés

**Affiliations:** ^1^Servicio de Cardiología, Hospital General Universitario Gregorio Marañón, Madrid, Spain; ^2^Instituto de Investigación Sanitaria Gregorio Marañón, Madrid, Spain; ^3^CIBER-CV, Madrid, Spain; ^4^Facultad de Medicina, Universidad Complutense, Madrid, Spain; ^5^Facultad de Ciencias Biomédicas y de la Salud, Universidad Europea, Madrid, Spain

**Keywords:** heart failure, sex, women, gender, prognosis

## Abstract

Heart failure (HF) affects 1–2% of the population in developed countries and ~50% of patients living with it are women. Compared to men, women are more likely to be older and suffer hypertension, valvular heart disease, and non-ischemic cardiomyopathy. Since the number of women included in prospective HF studies has been low, much information regarding HF in women has been inferred from clinical trials observations in men and data obtained from registries. Several relevant sex-related differences in HF patients have been described, including biological mechanisms, age, etiology, precipitating factors, comorbidities, left ventricular ejection fraction, treatment effects, and prognosis. Women have greater clinical severity of HF, with more symptoms and worse functional class. However, females with HF have better prognosis compared to males. This survival advantage is particularly impressive given that women are less likely to receive guideline-proven therapies for HF than men. The reasons for this better prognosis are unknown but prior pregnancies may play a role. In this review article we aim to describe sex-related differences in HF and how these differences might explain why women with HF can expect to survive longer than men.

## Introduction

Heart failure (HF) is an increasing global problem, with a current worldwide prevalence of more than 64 million cases, which means roughly 8.5 per 1,000 inhabitants (1). Although ~50% of patients with HF are women, sex-related differences within HF are poorly recognized, and understood. According to recent evidence, such differences may include biological mechanisms, epidemiology, pathogenesis, treatment response, quality of care, and prognosis.

The prevalence of HF increases with age, but this is particularly true in women, with a higher prevalence of HF in elderly women than in their male counterparts (2). While men more frequently suffer from HF as a consequence of ischemic heart disease (3–7), women with HF present with more frequent comorbidities such as hypertension, obesity and diabetes Besides, women with HF have higher left ventricular ejection fraction (LVEF) than men (8, 9). In fact, in acute decompensated HF, women tend to have preserved left ventricular systolic function almost twice as often as men (3, 10). HF management also has several sex-related differences, with women being less frequently studied for their underlying HF-etiology and their LVEF less often assessed than in men. In addition, women are less frequently treated with evidence-based drugs, even after adjustment for age, comorbidities, and LVEF (11, 12).

This review article focuses on the influence of sex in HF prognosis. Women are known to have a better prognosis than men in other cardiovascular conditions, including hypertension, aortic stenosis, and hypertrophic cardiomyopathy. Moreover, they typically adapt to those conditions with less chamber dilation, wall thinning, and better contractility than men (13). However, there are some exceptions where males do not fare worse than females such as Tako-tsubo syndrome or cardiac toxicity in alcoholic cardiomyopathy (14, 15).

## Biological Differences

It is widely known that male and female hearts and cardiovascular systems are different both at baseline and in response to insults (16). Women have smaller hearts, with lower end-diastolic pressures, and higher right ventricular ejection fraction, in spite of having similar LVEF (17). During exercise, women have greater increase in their end-diastolic volume as a compensation for their lower increase of LVEF compared to men. Over the years, women experience less deterioration in their contractile function (18).

When considering the causes for these differences, estrogens are obvious candidates. It has been demonstrated that cardiovascular risk increases when estrogen production ceases, being a strong argument in favor of their protective role. Moreover, the presence of estrogenic and androgenic receptors in cardiac tissue, which could influence the function of contractile proteins, has been proven. Furthermore, endogenous estrogens have been shown to be relatively protective from apoptosis and cell death in response to acute coronary ischemia, making women have greater myocardial salvage after successful reperfusion, smaller infarct sizes, less adverse cardiac remodeling, and higher preservation of left ventricular function (19–21).

Being an exclusive cause of female HF, peripartum cardiomyopathy is worth mentioning as an exception to favorable female hormonal influence. Several mechanisms such as myocarditis, autoimmune processes, and hemodynamic stress of pregnancy, all of them triggered by the hormonal context, have been studied as potential causes of this condition. As in other causes of HF in women, delayed diagnosis is not uncommon and is associated with more adverse outcomes. Worse prognosis is also related to the decrease of LVEF, the degree of left ventricular dilatation, obesity, and black race (22). Nevertheless, given its small prevalence, many questions remain about peripartum cardiomyopathy global prognosis compared to any other cause of HF (23).

Despite hormones playing a leading role, a single factor is unlikely to justify every difference found (24). This has led to the study of genetic predictors for cardiovascular disease, and for HF in particular, with no relevant findings to date For instance, women's Health Genome Study followed more than 19,000 women prospectively during a median of 12 years, showing no incremental capability to predict cardiovascular disease risk (25).

On the other hand, there is a tendency to think that the main cause of the prognostic benefit of women with HF is their higher frequency of diastolic HF. However, there is strong evidence against this thought. Although it is true that women have higher LVEF and therefore mid-range and systolic HF are less common in women than in men, several data have confirmed that women with HF have better survival than men irrespective of LVEF (5, 26). Female sex has also been proven to be an independent predictor of lower mortality in patients with HF with preserved ejection fraction (6). In addition, studies that included patients with systolic dysfunction showed that women live longer than men, even after adjustment for ischemic etiology and even when only patients with advanced systolic dysfuntion (LVEF < 20%) were considered (12, 27). In fact, LVEF seems to have less prognostic influence in women than in men (27, 28).

**Figure 1:**
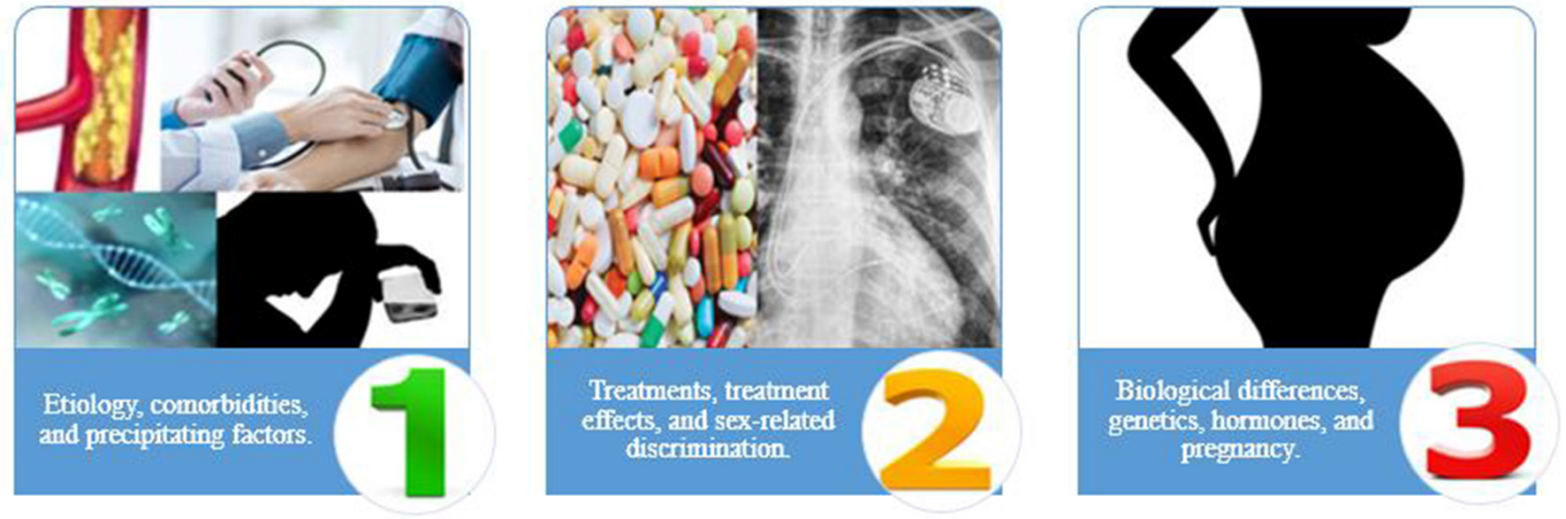
Determining factors of the differences in female heart failure syndrome.

In absence of other clear causes, sex related differences in HF prognosis have been associated with three additional mechanisms (Figure 1):

- Differences in etiology, prevalence of comorbidities, triggers, predisposing or precipitating factors.
- Treatments received and treatments effects.
- Previous pregnancies.

### Differences in Etiology and Comorbidities

The etiology of HF varies depending on sex, age, and race. Since many patients suffer from different conditions that might cause it, HF is often multifactorial. Ischemic heart disease, hypertension, valvular heart disease, and idiopathic dilated cardiomyopathy are the most frequent etiologies of HF, with different distribution according to sex (Table 1).

**Table 1:** Differences in heart failure etiologies.

**Male**	**Female**
Ischemic heart disease	Hypertension
Dilated cardiomyopathy	Valvular heart disease
Hypertrophic cardiomyopathy	Atrial fibrillation
Arrhythmogenic cardiomyopathy	

#### Hypertension

Hypertension is an important precursor of HF in general population. Global prevalence of hypertension is higher in women that in men, with this difference being more pronounced in the elderly (29). Multiple hypotheses try to explain this higher prevalence of hypertension in women, being the role of female sex hormones a known important contributing factor. While women are premenopausal, estrogens activate nitric oxide causing vasodilatation and reducing vascular stiffness (30). Moreover, ovarian hormones reduce plasma renin and angiotensin-converting enzyme activity (31). With the onset of menopause, the drop in estrogens levels is associated with an increased rigidity of the arterial wall due to collagen accumulation and elastin fragmentation, which leads to a two-fold greater risk of hypertension (29).

Regarding premenopausal women, oral contraceptive use could explain a certain trend to higher blood pressure, being associated with an increase in around 7–8 mmHg from baseline and almost double risk of hypertension compared with never-users (32, 33). Importantly, hypertensive women are more likely to develop left ventricular hypertrophy, diastolic dysfunction, and HF compared with men (3, 34). Levy and collaborators showed that the adjusted risk for HF development was about 2-fold in hypertensive men but 3-fold in hypertensive women compared to normotensive patients (35). Interestingly, they showed that hypertension could be causing 39% HF cases in men and 59% in women.

#### Ischemic Heart Disease

Ischemic heart disease is more common in men than in women (2). Even in the setting of acute coronary syndrome, women have less atherosclerotic burden and less plaque rupture than men (36, 37). Also in patients with chronic coronary artery disease, men have greater amount of coronary lesions, whereas women more frequently suffer from chest pain without obstructive coronary artery disease, which has been attributed to endothelial and microvascular dysfunction (38). Along with hypertension, ischemic heart disease is responsible for the largest proportion of the newly diagnosed cases of HF, being associated with a 52% of cases in the Framingham Heart Study (39). Importantly, ischemic heart disease is main cause of HF for men, whereas it plays a smaller role in the etiology of HF for women (3, 5, 40). However, large registries and clinical trials have shown that, in patients with coronary artery disease, women have higher risk of HF than men (41, 42). In the Pexelizumab in Conjunction With Angioplasty in Acute Myocardial Infarction (APEX-AMI) trial (42) female sex was an independent predictor of HF and cardiogenic shock. This difference in the risk of HF after a myocardial infarction persists not only throughout hospitalization but also during long-term follow-up (43). On the other hand, sex-bias has been identified in the diagnosis and treatment of ischemic heart disease. According to the Euro Heart Survey of Stable Angina (44, 45), women were less likely to undergo exercise electrocardiogram and coronary angiography than men. Women with ischemic heart disease were also less likely to be revascularized, and received antiplatelet treatment and statins less frequently, with a poorer control of cardiovascular risk factors including blood pressure and LDL-cholesterol. Interestingly, sex-related differences in HF prognosis are less marked in patients with ischemic etiology, and women survival benefit is lower in this context (7). Furthermore, men suffering from ischemic heart disease who bear an implantable cardioverter defibrillator suffered more ventricular arrhythmias and received more device therapies than women (46–48). This suggests that different degrees of susceptibility to arrhythmia triggering may explain differences in sudden cardiac death rates (49, 50).

#### Non-ischemic Dilated Cardiomyopathy

In recent years, there has been a broad advance in our knowledge of the genetic causes that justify the appearance of dilated cardiomyopathy, being most of the implicated genes autosomal dominant in transmission (51). Despite this common pattern, the hypothesis that sex may affect the penetrance of disease genes could explain why men have a slightly greater prevalence of dilated cardiomyopathy than women (52). Regarding prognosis, myocardial recovery is more common in women than in men, as is transplant-free survival (53). Several examples of female-protection have been described, as in the case of mutations in genes encoding for the sarcomere protein titin, found in ~25% of familial dilated cardiomyopathy cases, with male carriers suffering adverse events up to 10 years earlier than females (54). Whether these differences are caused by variances in factors such as penetrance, expressivity, modifier genes, or environmental factors, remain unknown (53).

#### Atrial Fibrillation

Women with atrial fibrillation (AF) have larger left atrial volume index and lower emptying fraction than men (55). While AF increases the risk of HF in women, this association has not been clearly established for men (56). In addition, in females AF is associated with greater risk for adverse clinical outcomes, particularly HF hospitalization (57).

#### Other Cardiomyopathies

See Table 2.

**Table 2:** Sex-related differences in heart failure etiology and its implication in prognosis.

**Other causes**		**Males**	**Females**	**References**
Valvular heart disease	Mitral regurgitation	Equal prevalence	Frequent underdiagnosis and delayed valvular interventions. Less mitral valve repair, worse outcomes associated with replacement. Higher probability of recurrent HF after surgery. Similar outcomes after Mitraclip.	(58–60)
	Aortic stenosis	Equal prevalence and similar prognostic implications for both sexes. More frequently referred for surgery.	Higher prevalence of paradoxical low flow- low gradient stenosis. More frequent concomitant significant mitral disease. Similar survival rates after surgery. Lower all-cause mortality after TAVR.	(61–64)
	Tricuspid regurgitation		Higher prevalence. Similar results in isolated surgery, but poorer perioperative outcomes when combined with coronary artery bypass surgery.	(65, 66)
Other cardiomyopathies	Hypertrophic cardiomyopathy	Higher prevalence (2:1 predominance in males). More hypertrophy and fibrosis. More ventricular arrhythmias	Worse symptoms Higher all-cause mortality	(67, 68)
	Arrhythmogenic cardiomyopathy	Higher prevalence (approximate ratio of 3:1). Higher mortality rate and sudden cardiac death.		(69, 70)
	Restrictive cardiomyopathy	Male predominance in mutant and Wild-type transthyretin amyloid. More frequent Cardiac involvement in sarcoidosis.	Higher occurrence of endomyicardial fibrosis, but better survival. No sex differences for hyper-eosinophilic syndrome, scleroderma or carcinoid heart disease.	(52, 71)

*TAVR, transcatheter aortic valve replacement*.

### Differences in Treatment Administration and Response

Women have been historically underrepresented in HF clinical trials and, to a lesser amount, in registries. Moreover, many data come from *post-hoc* analyses and registries, with their inherent bias (26). This has limited our understanding of the efficacy of HF treatment in women (72). Moreover, it has been shown that women are less likely to receive guideline-proven HF therapies than men, and more frequently receive suboptimal doses (11, 40). However, adherence to HF treatments is higher in women than in men (73, 74).

#### Drugs to Treat HF With Reduced Ejection Fraction

**Figure 2:**
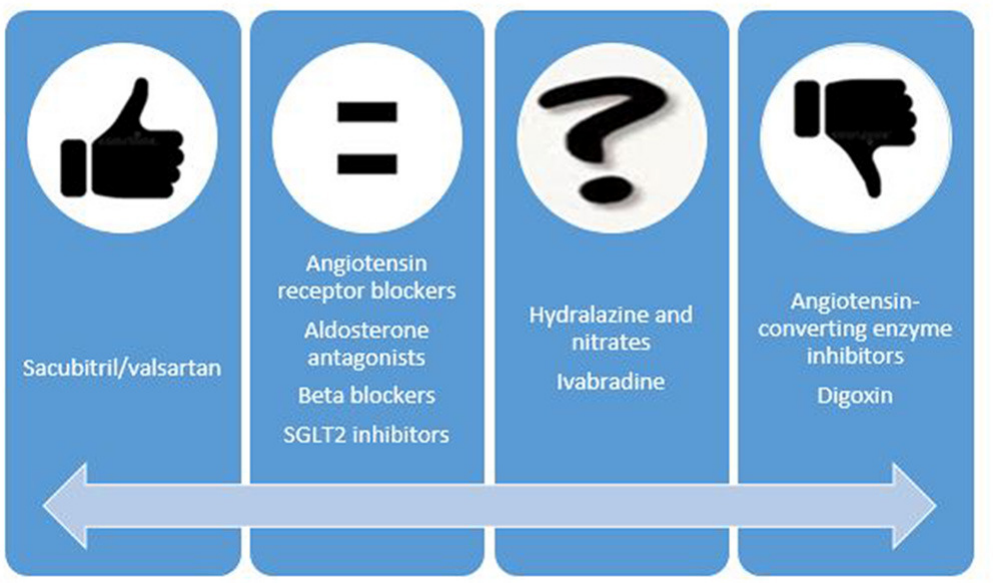
Possible sex-related differences in the benefit of heart failure drugs. Thumb up means data that suggest higher benefit in women than in men. Thumb down means the opposite.

Women with HF and reduced ejection fraction receive significantly less furosemide than men, both at admission and during hospitalizations (12, 75). Regarding angiotensin-converting enzyme (ACE) inhibitors, the benefit for women may not be as great as for men, with particular doubts concerning its value in women with still asymptomatic LV systolic dysfunction (76, 77). However, this is probably related with limited power due to the low representation of women in studies (78). Conversely, the effect of angiotensin receptor blockers (ARB) seems to be similar in both sexes (79). Sacubitril/valsartan has a similar tolerability in men and women with more frequent functional class improvement and greater reduction in the risk of HF hospitalization in women than in men (80, 81). The data regarding hydralazine and isosorbide dinitrate in females are extremely scarce, being particularly surprising given that this combination is frequently used to treat HF during pregnancy, when ACE inhibitors and ARBs are contraindicated. Besides, spironolactone and eplerenone improve survival in symptomatic systolic HF in men and women (82–84) (Figure 2).

On the other hand, betablockers improve outcomes in women, even though the main benefits in most studies were related to the reduction in hospitalizations (85–87). At any rate, meta-analyses data have confirmed that the effect of betablockers in mortality reduction is similar in both sexes (76). Less than 25% of patients in ivabradine trials were women. Despite the limited evidence, there is no reason to think that their main benefit, the reduction in hospital admissions, is different in men and women (88). In contrast, a previous study yielded worrying results regarding digoxin use in women due to its possible association with an increased risk of death. Digoxin use and dosage should, therefore, be very cautious in women (89). Finally, sodium glucose co-transporter 2 (SGLT2) inhibitors have demonstrated benefits in terms of cardiovascular mortality and especially in lowering the risk of HF hospitalization (90) and the benefit seems to be similar in women and men (91).

#### Devices

Women are less often considered eligible for implantable cardioverter defibrillator (ICD) implantation, and even after adjustment for potential confounders, women are 40% less likely to receive ICD therapy than men (92–94). This is not justified by a lower efficacy in this subgroup, since previous studies have shown similar ICD effectiveness in both sexes (48).

Regarding resynchronization therapy (CRT), women are, once again, significantly less likely to undergo CRT implant compared to men despite its demonstrated greater benefit (95). Among patients enrolled in the Multicenter Automatic Defibrillator Implantation Trial with Cardiac Resynchronization Therapy (MADIT-CRT) trial, women treated with CRT experienced greater reductions in the combined endpoint of HF or death and had more reverse cardiac remodeling (96). Similar findings were found in the Multicenter InSync Randomized Clinical Evaluation (MIRACLE) study, with woman having less occurrence of HF or death than men (97).

#### Ventricular Assist Devices

Left ventricular assist devices (LVAD) are mainly used in men, with only 21–33% being implanted in women (98). There was an initial concern that women had increased mortality and risk of bleeding or neurologic events compared with men (99, 100). However, recent evidence has shown no significant sex-related differences in terms of infections, bleeding, or device malfunction (26, 101, 102). Moreover, survival with LVAD has improved for both women and men with no differences in mortality (98, 103). The main persistent limitation is the female higher risk of neurologic events (101), even though some authors have blamed the differences in axial vs. centrifugal continuous flow and the dissimilarities in anticoagulation treatment as potential explanations for these differences. In fact, the latest models such as HeartMate 3 have no sex-related difference in stroke risk (104).

#### Heart Transplantation

Heart transplantation provides the best opportunity of quality and quantity of life for eligible patients with advanced HF (105). However, women are significantly less frequently transplanted, being approximately a quarter of total transplants (106). This has a multifactorial explanation, but age is likely an important factor since older age decreases eligibility for heart transplantation (98). Women also have a higher likelihood to be sensitized with antibodies, although few women are not referred to transplant for this reason (107). Among patients in heart transplant waitlist, women have worse prognosis, probably because only those with more severe forms reach that list, but also due to the lower rates of mechanical circulatory support despite similar INTERMACS status. That could also explain why this higher mortality risk only applies for women listed high priority, whereas those listed as low priority have similar or even better prognosis than men (108). Survival after heart transplantation is better in women with a median survival of 11.5 years as opposed to 10.5 years for men (105). Conversely, they admit worse quality of life and worse functional class, with more frequent depression not only early but also later after transplantation (109). Regarding long-term associated diseases, men recipients suffer significantly more frequent post-transplant malignancy (110), which is not only related to sex-specific cancers, as this risk remains after exclusion of prostate, breast and cervical cancer (111). On the other hand, although some previous evidences have suggested that women have higher risk of antibody-mediated rejection, which is supposed to subsequently increase their risk of cardiac allograft vasculopathy, in fact coronary vasculopathy is also less frequent in women, being an important difference to bear in mind during follow-up (112, 113).

Finally, it is worth mentioning that sex is an important fact when it comes to deciding the recipient for a particular donor, as some studies have highlighted the prognostic importance of donor/recipient sex-mismatch (114). Particularly, male recipients have been found to have a worse prognosis after a sex mismatch transplant, whereas women seem to do similarly when they receive a male allograft. Although some anatomical, physiological, and immunological facts have been suggested, the reasons for this interaction remain unknown (115).

### Pregnancies

Women's bodies experience a non-pathological period of strong changes for the anatomy and physiology of the heart: pregnancy. This carries a huge increase in ventricular volumes, cardiac output and ventricular hypertrophy as well as a significant decrease in vascular resistance due to vasodilatation and the interposition of a low resistance circuit such as the placenta (116). This cardiovascular remodeling, as well as the ability to adapt volume overcharge, have been suggested to be a sort of training for the heart, which could represent a benefit in terms of preventing HF or improving its global prognosis if it occurs. Furthermore, persisting fetal male cells have been found in the hearts of women with previous pregnancies. This microchimerism has been hypothesized to be beneficial for the mother's heart, and even lead to a better tolerance to the graft in case of transplant (117). Although more studies are required to quantify the benefits of previous pregnancies in HF outcomes, a previous series including 756 females with HF found an association between the number of previous gestations and better 1-year survival (HR 0.878, 95% CI: 0.773–0.997, *P* = 0.045) (118)

## Discussion

As a result of all previous explained differences, HF syndrome seems to have several distinctive features in women. They have greater clinical severity of HF, evidenced by worse functional class and higher prevalence of symptoms and signs, with more frequent edemas, murmurs, rales, jugular venous distension and gallop (5, 53, 119). They also tend to have more comorbidities such as anemia, iron deficiency, renal disease and thyroid abnormalities, while frailty sex-differences have not been extensively analyzed in HF patients (120). As a consequence, women with HF have significantly lower global quality of life and higher ratings for anxiety and worse social activity (121, 122). Previous articles that have studied the differences in quality of life in HF defined social health as the sum of social function, social life satisfaction, and intimacy (120). Riedinger et al., using the Functional Status Questionnaire, found that women had worse general life satisfaction and social health than men (121). We could speculate that as women usually have more social activities than men, including visiting relatives and participating in community activities, when they reduce these activities due to HF-related symptoms they might have a worse social life satisfaction. Besides, they are also more likely to suffer from depression than men (123).

Whether this greater severity translates into differences in HF hospitalizations was classically controversial, but nowadays most studies agree that after adjustment for relevant covariates, women with HF are less prone to cardiovascular or all-causehospitalizations than men (5). Thereby, male sex is an independent risk factor for all-cause admissions after HF diagnosis (124). Particularly, recent evidences shows that women have a 13% lower adjusted risk of HF hospitalization, with this risk being also lower in women with low LVEF (38). However, once admitted for HF, women tend to have an increased length of stay, although this does not affect to in-hospital mortality, which is comparable among both sexes (125). A large multicenter registry confirmed that despite differences in baseline characteristics, women and men with both reduced and preserved LVEF have similar in-hospital mortality and risk factors predicting death (126).

Survival after the onset of HF has been improving in both sexes in recent decades (127). Regarding sex-differences in mortality, in the vast majority of trials and registries women with HF have better age-adjusted survival rate than men (5, 40, 118, 128). They have a lower risk of death irrespective of cause of HF and of comorbidities (7, 40). This benefit is more apparent when the etiology is unrelated to ischemia, as women with HF related to non-ischemic diseases have significantly better survival than men with or without coronary artery disease as their main cause of HF. (7, 129). Furthermore, LVEF has lower prognostic influence in women than in men (28, 130).

On the other hand, women with HF included in the CHARM (Candesartan in Heart Failure: Assessment of Reduction in Mortality and morbidity) program had lower adjusted risk not only of cardiovascular death but also of non-cardiovascular death. Particularly for the first group, that risk was lower for the two main cardiovascular types of death related to HF, pump failure and sudden death (38). Other studies have also shown that male sex is in fact one of the main predictors for sudden cardiac death (131). Notwithstanding, given that the reduction of mortality is comparable for both, it is not possible to clarify if the benefit is mainly due to electrical stability or the pump function itself. More studies regarding this sex differences in mortality and its causes are needed.

## Conclusions

HF represents a major global health issue with important sex-related differences in several aspects that include epidemiology, natural history, clinical manifestations, effects of therapy, and prognosis. Women are underrepresented in clinical studies. Women peculiarities also include genetics, comorbidities, hormones, and pregnancy. Compared to men, women are more likely to be older and suffer hypertension, valvular heart disease, and non-ischemic cardiomyopathy. Women have greater clinical severity of HF, with more symptoms and worse functional class. However, females with HF have better prognosis compared to males. This survival advantage is particularly impressive given that women are less likely to receive guideline-proven therapies for HF than men.

### Future Perspectives

Understanding the underlying sex-related differences within HF may improve the management of HF by presenting more targeted options for personalized medicine.

## Author Contributions

AP: conceptualization, writing—original draft, and writing—review & editing. MM-S: conceptualization, investigation, resources, writing—review & editing, supervision, and project administration. All authors contributed to the article and approved the submitted version.

## Conflict of Interest

The authors declare that the research was conducted in the absence of any commercial or financial relationships that could be construed as a potential conflict of interest.
